# 丹参酮类化合物对SPC-A-1细胞的生长抑制及其构效关系探讨

**DOI:** 10.3779/j.issn.1009-3419.2011.01.02

**Published:** 2011-01-20

**Authors:** 华月 石, 青 张, 慧 李, 婷 储, 辉 金, 声俊 毛

**Affiliations:** 1 610041 成都，四川大学华西药学院 West China School of Pharmacy, Sichuan University, Chengdu 610041, China; 2 610072 成都，四川省医学科学院，四川省人民医院 Sichuan Academy of Medical Sciences and Sichuan Provincial People's Hospital, Chengdu 610072, China

**Keywords:** 丹参酮类化合物, 肺肿瘤, 构效关系, Tanshinone, Lung neoplasms, Structure-activity relationship

## Abstract

**背景与目的:**

大量研究表明丹参酮类化合物具有体外抗肿瘤作用，但很少有人综合研究丹参酮类化合物对同一种肿瘤细胞的作用情况。本文旨在比较4种丹参酮类化合物对SPC-A-1细胞的增殖抑制作用，并探讨其结构与细胞毒性之间的关联性。

**方法:**

采用改良MT法测定不同浓度的丹参酮类化合物与细胞共培养预定时间（24 h、48 h和72 h）后对SPC-A-1细胞的增殖抑制作用；倒置显微镜下观察不同药物处理对于SPC-A-1细胞的形态学影响。

**结果:**

丹参酮类化合物均能有效抑制SPC-A-1细胞增殖，其抑制作用呈明显的时间和剂量依赖性。二氢丹参酮Ⅰ、丹参酮Ⅰ、丹参酮ⅡA、隐丹参酮作用24 h的IC_50_值分别为2.77 μg/mL、6.01 μg/mL、 > 10 μg/mL和 > 10 μg/mL；作用48 h的IC_50_值分别为1.80 μg/mL、4.04 μg/mL、8.12 μg/mL、8.71 μg/mL；作用72 h的IC_50_值分别为1.36 μg/mL、1.69 μg/mL、3.81 μg/mL、7.35 μg/mL。

**结论:**

4种丹参酮类化合物均对SPC-A-1细胞具有明显的增殖抑制作用，作用强度大小依次为二氢丹参酮Ⅰ、丹参酮Ⅰ、丹参酮ⅡA、隐丹参酮，提示A环为芳环时可增强细胞毒性，C环的呋喃环结构可能影响其细胞毒性，其具体作用机理尚有待探讨。

## Abstract (en)

**Background and objective:**

Numerous studies have shown that Tanshinones have anti-tumor effects *in* *vitro*, but few studies were focusing on the anti-tumor activity of Tanshinones against one special cancer cell line. The aim of this study is to investigate the growth inhibition effect of four Tanshinones on SPC-A-1 cell line and the relationship between their structures and cytotoxicity.

**Methods:**

The modifed MT assay was adopted to measure the inhibition effect of Tanshi nones on SPC-A-1 cells at different concentrations at 24 h, 48 h and 72 h, and the changes of cell morphology were observed by inverted phase contrast microscope.

**Results:**

Tanshinones could inhibit the proliferation of SPC-A-1 cells effectively, and their cytotoxicities on SPC-A-1 cells are all in concentration-dependent and time-dependent manners. The IC_50_ of dihydro Tanshinone Ⅰ, Tanshinone Ⅰ, Tanshinone ⅡA and Cryptotanshinone at 24 h were 2.77 μg/mL, 6.01 μg/mL, over 10 μg/mL and over 10 μg/mL, at 48 h were 1.80 μg/mL, 4.04 μg/mL, 8.12 μg/mL, 8.71 μg/mL, at 72 h were 1.36 μg/mL, 1.69 μg/mL, 3.81 μg/mL, 7.35 μg/mL, respectively.

**Conclusion:**

All of the four Tanshinones have proliferation inhibitory effects on SPC-A-1 cell line, among which the Dihydrotanshinone Ⅰ is the most active one, followed by Tanshinone Ⅰ, Tanshinone ⅡA and Cryp totanshinone subsequently. The results showed that the structure of aromatic ring A could enhance the cytotoxicity and the structure of furan ring C would influence the cytotoxicity, but the mechanism is still remained to be further investigated.

肺癌在我国已占据恶性肿瘤发病率及死亡率的第1位，调查数据显示，我国肺癌的发病率和死亡率一直呈上升趋势。尽管近20年来肺癌的治疗有了很大的进展，但肺癌患者的预后却无明显提高，5年生存率仅10%-15%，而其它肿瘤的平均5年生存率能达到62%-97%^[1, 2]^。肺癌的流行及其严重危害已使其治疗药物成为临床不可缺少且地位极其重要的一大类药物。因此，寻找新的抗癌药物对肺癌的治疗具有重大意义。

丹参为唇形科鼠尾草属植物（Salvia miltiorrhiza bunge）的干燥根部，具有祛瘀止痛、活血调经、养心除烦等功效，入药历史悠久。丹参酮（Tanshinone, Tan）作为丹参根部的乙醚或乙醇提取物，是丹参的主要有效部位。按其不同的化学结构可分为丹参酮Ⅰ、丹参酮ⅡA、丹参酮ⅡB、隐丹参酮、二氢丹参酮Ⅰ等15种成分（图 1）。近年来大量研究^[3-6]^证实，丹参酮类化合物具有广泛的抗肿瘤活性，是一种有前途的抗癌药物。本研究以人肺腺癌细胞株SPC-A-1为研究对象，考察丹参酮类化合物（丹参酮Ⅰ、丹参酮ⅡA、隐丹参酮、二氢丹参酮Ⅰ）对其生长抑制作用，并比较四者对SPC-A-1细胞株的药效学差异，为探讨丹参酮类化合物细胞毒性与其结构之间的关系以及开发用于治疗肺癌的丹参酮制剂提供实验依据。

**1:**
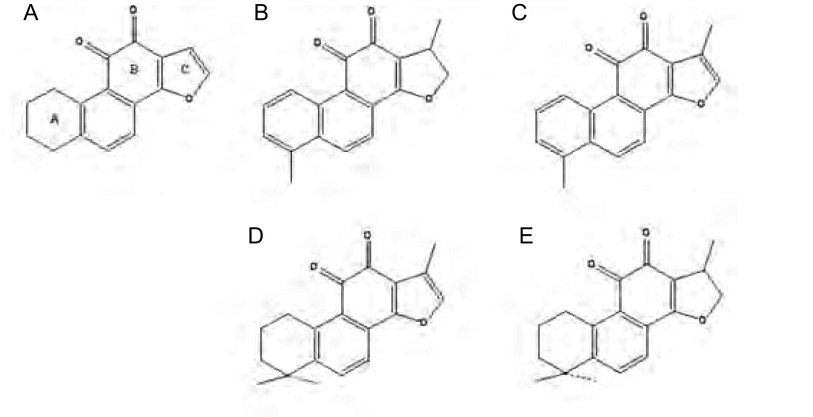
丹参酮类化合物化学结构。A：丹参酮类化合物基本结构；B：二氢丹参酮Ⅰ；C：丹参酮Ⅰ；D：丹参酮ⅡA；E：隐丹参酮。 Chemical structures of Tanshinones. A: basic structure of Tanshi- nones; B: Dihydrotanshinone Ⅰ; C: Tanshinone Ⅰ; D: TanshinoneIIA; E: Cryptotanshinone.

## 1 材料和方法

### 1.1 材料

#### 1.1.1 试剂与药品

RPMI-1640培养基为美国Gibco公司产品；标准新生牛血清为郑州伯安生物工程有限公司产品；四甲基偶氮唑蓝（MTT）为BIOSHARP公司产品；二甲基亚砜（DMSO）分析纯为天津市博迪化工有限公司产品；十二烷基硫酸钠（SDS）分析纯为广东光华化学厂有限公司产品；亚砷酸注射液（H_3_AsO_3_）为哈尔滨伊达药业有限公司产品，实验前用RPMI-1640培养液稀释成2 μg/mL的药液；丹参酮ⅡA为西安天丰生物科技有限公司产品，由本实验室精制，纯度达99.2%（HPLC）；丹参酮Ⅰ、二氢丹参酮Ⅰ、隐丹参酮均为成都曼思特生物科技有限公司产品；丹参酮类化合物均用DMSO溶解（DMSO终浓度为0.03%，一般认为DMSO≤0.1%时，不引起细胞生物学形态改变），配制成1.7 mg/mL的储备液，过滤除菌，于4 ℃条件下避光保存备用。

#### 1.1.2 细胞株

人肺腺癌SPC-A-1细胞株由生物治疗国家重点实验室提供，用含10%灭活标准新生牛血清、100 U/mL青霉素、100 μg/mL链霉素的RPMI-1640培养液，于37 ℃、5%CO_2_、饱和湿度的培养箱中培养，每2-3天传代1次。实验用对数生长期细胞。

### 1.2 方法

#### 1.2.1 MTT法检测细胞生长抑制率

取对数生长期的SPC-A-1细胞，消化制成单细胞悬液后计数，调整其密度为2×10^4^个/mL，接种于96孔板中，每孔100 μL；接种细胞后，于37 ℃、5%CO_2_、饱和湿度的培养箱中培养4 h后给药。以等体积的亚砷酸（2 μg/mL）作为参比药物组，不加药的细胞组作为空白对照组，RPMI-1640培养液组作为空白组用以调零。给药组分别加入不同浓度的丹参酮ⅡA、丹参酮Ⅰ、二氢丹参酮Ⅰ和隐丹参酮，给药浓度分别为2.0 μg/mL、4.0 μg/mL、6.0 μg/mL、8.0 μg/mL、10.0 μg/ mL，每个浓度设6个复孔，每孔20 μL。分别于培养24 h、48 h、72 h后，加入5 mg/mL MTT液（每孔20 μL），于37 ℃、5%CO_2_孵箱中继续培养。4 h后按照文献^[7]^中的方法，加入由异丁醇、浓盐酸、十二烷基硫酸钠（SDS）配制的甲瓒裂解液（100 μL/孔），于37 ℃、5%CO_2_培养箱中孵育过夜至甲瓒全部溶解，微孔板恒温震荡仪震荡10 min，使完全混匀。酶标仪570 nm（参比波长630 nm）处测各孔光密度（optical density, OD）值，以该复孔的平均值作为该组细胞的OD值，实验重复3遍，结果取其平均值，并按公式计算细胞生长抑制率。细胞增殖抑制率计算公式为：

抑制率=（1-实验组平均OD值/空白对照组平均OD值）×100%。

#### 1.2.2 倒置显微镜观察细胞形态生长情况

在加药实验过程中，分别于24 h、48 h和72 h观察丹参酮化合物组（4.0 μg/mL）、参比药物组（2 μg/mL）以及空白对照组细胞的生长情况。

#### 1.2.3 统计学处理

采用SPSS 17.0软件进行统计分析，数据以Mean±SD表示，组间比较采用*One way ANOVA*检验，*P* < 0.05为有统计学差异。

## 2 结果

### 2.1 丹参酮类化合物对SPC-A-1细胞的增殖抑制作用

SPC-A-1细胞经4种药物处理后，与空白对照组相比，其OD值均有不同程度的下降，且随着药物浓度的增加及作用时间的延长，OD值下降趋势明显（表 1-表 4）。其中，二氢丹参酮Ⅰ组、丹参酮Ⅰ组中细胞的OD值下降较明显，2.0 μg/mL处理24 h后，其OD值与对照组相比有统计学差异；二氢丹参酮Ⅰ组、丹参酮Ⅰ组和丹参酮ⅡA在2.0 μg/mL处理24 h后表现出增殖抑制作用。隐丹参酮组起效较慢，6.0 μg/mL作用24 h才表现出增殖抑制作用，且2.0 μg/mL隐丹参酮分别作用48 h和72 h时抑制作用均不明显，OD值与对照组相比均无统计学差异。与参比药物组相比，4种药物对细胞的增殖抑制作用均与亚砷酸作用趋势一致，且4.0 μg/mL二氢丹参酮Ⅰ、4.0 μg/mL丹参酮Ⅰ、8.0 μg/mL丹参酮ⅡA、10.0 μg/mL隐丹参酮作用72 h后的抑制率（分别为83.40%、72.57%、62.37%、69.96%）与2 μg/ mL亚砷酸作用72 h（65.22%）的抑制率相当或更高。4种药物（二氢丹参酮Ⅰ、丹参酮Ⅰ、丹参酮ⅡA、隐丹参酮）作用24 h后IC_50_值分别为2.77 μg/mL、6.01 μg/mL、 > 10.00 μg/mL和 > 10.00 μg/mL；作用48 h的IC_50_值分别为1.80 μg/ mL、4.04 μg/mL、8.12 μg/mL、8.71 μg/mL；作用72 h的IC_50_值分别为1.36 μg/mL、1.69 μg/mL、3.81 μg/mL、7.35 μg/mL，可见4种药物中二氢丹参酮Ⅰ起效浓度最低，其次为丹参酮Ⅰ、丹参酮ⅡA和隐丹参酮，且其抑制率均呈明显的时间正相关性（表 5）。

**1:** 不同浓度二氢丹参酮Ⅰ在不同时间点对SPC-A-1细胞的生长抑制作用（Mean±SD） Growth inhibition effect of Dihydrotanshinone Ⅰ over a range of concentrations on SPC-A-1 cell line at different times(Mean±SD)

Concentration（*μ*g/mL）	24 h			48 h			72 h	
	OD	Inhibitory rate (%)		OD	Inhibitory rate (%)		OD	Inhibitory rate (%)
Control	0.275±0.011	—		0.458±0.010	—		0.565±0.003	—
2.0	0.143±0.010^*^	48.09		0.231±0.034^*^	49.54		0.229±0.044^*^	59.47
4.0	0.125±0.010^*^	54.64		0.086±0.009^*^	81.20		0.094±0.023^*^	83.40
6.0	0.124±0.018^*^	55.01		0.056±0.003^*^	87.71		0.033±0.005^*^	94.12
8.0	0.119±0.016^*^	56.56		0.039±0.006^*^	91.53		0.026±0.005^*^	95.40
10.0	0.078±0.013^*^	71.49		0.031±0.004^*^	93.34		0.012±0.004^*^	97.92
OD: optical density. ^*^P < 0.05, compared with the control group.								

**2:** 不同浓度丹参酮Ⅰ在不同时间点对SPC-A-1细胞的生长抑制作用（Mean±SD） Growth inhibition effect of TanshinoneⅠover a range of concentrations on SPC-A-1 cell line at different times (Mean±SD)

Concentration （*μ*g/mL）	24 h			48 h			72 h	
	OD	Inhibitory rate (%)		OD	Inhibitory rate (%)		OD	Inhibitory rate (%)
Control	0.275±0.011	—		0.458±0.010	—		0.565±0.003	—
2.0	0.241±0.022^*^	12.20		0.385±0.039^*^	15.95		0.285±0.050^*^	49.60
4.0	0.167±0.017^*^	39.44		0.146±0.009^*^	68.21		0.155±0.007^*^	72.57
6.0	0.125±0.004^*^	54.55		0.130±0.004^*^	71.76		0.144±0.011^*^	74.50
8.0	0.122±0.013^*^	55.65		0.129±0.006^*^	71.82		0.135±0.003^*^	76.06
10.0	0.084±0.012^*^	69.49		0.114±0.011^*^	75.20		0.118±0.016^*^	79.07
^*^P < 0.05, compared with the control group.								

**3:** 不同浓度丹参酮ⅡA在不同时间点对SPC-A-1细胞的生长抑制作用（Mean±SD） Growth inhibition effect of Tanshinone ⅡA over a range of concentrations on SPC-A-1 cell line at different times (Mean±SD)

Concentration （*μ*g/mL）	24 h			48 h			72 h	
	OD	Inhibitory rate (%)		OD	Inhibitory rate (%)		OD	Inhibitory rate (%)
Control	0.290±0.009	—		0.460±0.06	—		0.473±0.012	—
2.0	0.259±0.020	10.54		0.314±0.028^*^	31.72		0.310±0.029^*^	34.46
4.0	0.225±0.028^*^	22.45		0.286±0.049^*^	37.81		0.211±0.034^*^	55.44
6.0	0.220±0.030^*^	24.01		0.245±0.017^*^	46.71		0.204±0.044^*^	56.98
8.0	0.209±0.018^*^	27.81		0.238±0.020^*^	48.23		0.178±0.020^*^	62.37
10.0	0.1940±0.023^*^	33.07		0.209±0.087^*^	54.54		0.113±0.007^*^	76.22
^*^P < 0.05, compared with the control group.								

**4:** 不同浓度隐丹参酮在不同时间点对SPC-A-1细胞的生长抑制作用（Mean±SD） Growth inhibition effect of Cryptotanshinone over a range of concentrations on SPC-A-1 cell line at different times (Mean±SD)

Concentration （*μ*g/mL）	24 h			48 h			72 h	
	OD	Inhibitory rate (%)		OD	Inhibitory rate (%)		OD	Inhibitory rate (%)
Control	0.290±0.009	—		0.458±0.010	—		0.467±0.027	—
2.0	0.319±0.012^*^	—		0.454±0.036	0.76		0.425±0.030	7.80
4.0	0.292±0.011	—		0.358±0.018^*^	21.90		0.353±0.028^*^	23.43
6.0	0.221±0.016^*^	23.58		0.322±0.026^*^	29.60		0.309±0.156^*^	32.92
8.0	0.216±0.007^*^	25.56		0.234±0.024^*^	48.83		0.215±0.031^*^	53.36
10.0	0.199±0.006^*^	31.43		0.199±0.024^*^	56.47		0.139±0.034^*^	69.96
^*^P < 0.05, compared with the control group.								

**5:** 丹参酮类化合物对SPC-A-1的IC_50_值（24 h、48 h和72 h） IC_50_ of Tanshinones on SPC-A-1 cells at 24 h, 48 h and 72 h

Drug	IC_50_（μg/mL）		
	24 h	48 h	72 h
Dihydrotanshinone Ⅰ	2.77	1.80	1.36
Tanshinone Ⅰ	6.01	4.04	1.69
Tanshinone ⅡA	> 10	8.12	3.81
Cryptotanshinone	> 10	8.71	7.35

### 2.2 倒置相差显微镜观察细胞生长形态

SPC-A-1细胞培养48 h后，空白对照组可见贴壁生长、细胞清晰，胞质饱满，核膜、核仁轮廓明显，相邻细胞生长融合成片（图 2A）；2 μg/mL亚砷酸组（图 2B）可见视野中大部分细胞失去贴壁特性，细胞逐渐由贴壁而脱落，细胞皱缩、体积变小、变形，呈现出明显的增殖抑制作用与细胞凋亡特征；二氢丹参酮Ⅰ组（图 2C）和丹参酮Ⅰ组（图 2D）中的细胞形态与亚砷酸组相似，多数细胞由贴壁而脱落，出现细胞变小、皱缩和变形现象，且与空白对照组相比视野中细胞数明显减少；丹参酮ⅡA组（图 2E）和隐丹参酮组（图 2F）中，可见部分细胞由贴壁而脱落，呈现细胞皱缩、变小和变形的状态，与空白对照组相比视野中细胞数明显减少，但较二氢丹参酮Ⅰ组（图 2C）和丹参酮Ⅰ组（图 2D）视野中细胞数较多。该结果表明，四种丹参酮类化合物均能抑制SPC-A-1细胞生长，诱导其凋亡，其中二氢丹参酮Ⅰ作用强度最强，丹参酮Ⅰ和丹参酮ⅡA次之，隐丹参酮的作用强度则相对较弱，与MTT检测的结果一致。

**2:**
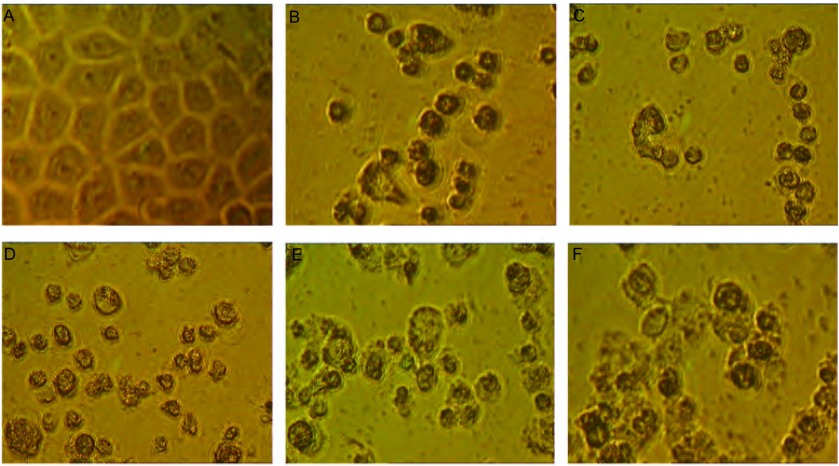
SPC-A-1细胞培养48 h的倒置显微镜图。A：未经药物处理的细胞；B：2.0 *μ*g/mL亚砷酸组；C：4.0 *μ*g/mL二氢丹参酮Ⅰ组；D：4.0 *μ*g/mL丹参酮Ⅰ组；E：4.0 *μ*g/mL丹参酮ⅡA组；F：4.0 *μ*g/mL隐丹参酮组。 Cell morphology observed by inverted phase contrast micro- scope after incubated for 48 h. A: no treatment; B: treated with 2 *μ*g/mL arsenious acid; C: treated with 4.0 *μ*g/mL dihydrotanshinone Ⅰ; D: treated with 4.0*μ*g/mL tanshinone Ⅰ; E: treated with 4.0 *μ*g/mL tanshi- none IIA; F: treated with 4.0 *μ*g/mL cryptotanshinone.

## 3 讨论

丹参酮是中药丹参的主要脂溶性成分，目前已有大量报道其在体外能对抗多种肿瘤细胞，作用机制主要涉及抑制肿瘤细胞增殖^[8]^、诱导细胞凋亡与分化、抑制细胞侵袭及转移^[9]^等方面。2008年，Lee^[10]^观察研究了二氢丹参酮Ⅰ、丹参酮Ⅰ、丹参酮ⅡA及隐丹参酮对HepG2细胞的毒性作用与GSH/GSSG（还原型谷胱甘肽/氧化型谷胱甘肽）比例变化之间的关系，结果表明二氢丹参酮Ⅰ及丹参酮Ⅰ诱导的细胞毒性作用与GSH/GSSG比例变化呈明显的正相关性，而丹参酮ⅡA及隐丹参酮则无此相关性；Wayne^[11]^用此4种丹参酮化合物处理*p53*基因表型不同的3株肝癌细胞系以及Pgp过表达的R-HepG2细胞，结果发现二氢丹参酮Ⅰ及丹参酮Ⅰ对4种肝癌细胞均有较好的增殖抑制作用，而隐丹参酮和丹参酮ⅡA则对Pgp过表达的R-HepG2更有效，其中隐丹参酮能有效抑制Pgp介导的药物外排，丹参酮ⅡA与阿霉素联用则表现出最强的药物协同作用。这些研究结果表明不同的丹参酮化合物的抗肿瘤活性存在分子机制上的差异，提示这可能与其化学结构不同有关。

本研究以人肺腺癌细胞株SPC-A-1作为细胞模型，研究比较了丹参酮类4种化合物（丹参酮Ⅰ、丹参酮ⅡA、隐丹参酮、二氢丹参酮Ⅰ）的细胞毒性作用，结果表明4种化合物均能有效抑制细胞生长，且抑制作用呈明显的时间、浓度依赖性。通过比较4种化合物作用24 h、48 h、72 h后的IC_50_值可发现，4种化合物的细胞增殖抑制作用强度依次为二氢丹参酮Ⅰ、丹参酮Ⅰ、丹参酮ⅡA、隐丹参酮，此结果与叶因涛等^[12]^在宫颈癌HeLa细胞研究方面实验结论基本一致，而在肺癌研究领域还无相关报道，本研究对于开发用于治疗肺癌的丹参酮制剂有重要意义。丹参酮Ⅰ、丹参酮ⅡA、隐丹参酮及二氢丹参酮Ⅰ均为丹参二萜醌类化合物，其结构差异主要表现在A环与C环的不同（图 1）。本实验结果表明，A环为芳环的化合物（二氢丹参酮Ⅰ和丹参酮Ⅰ）的细胞毒性明显强于A环为脂环的化合物（丹参酮ⅡA和隐丹参酮），其原因可能是当A环为芳环时，可影响整个分子的三维图像，表现为使B、C两个环平面之间的两面夹角减小，从而增强化合物细胞毒性^[13]^。对于呋喃环和醌类产生细胞毒性的作用机制，长期以来的观点是其能产生自由基而引起DNA损伤。2008年，Zhang^[14, 15]^否认了这一假设，其研究结果显示丹参酮ⅡA的呋喃氧并不产生自由基，而是通过与DNA双螺旋中腺嘌呤上的N形成氢键，改变DNA构型，导致RNAPII磷酸化并降解，激活*p53*基因，从而产生诱导细胞凋亡的作用；并进一步认为，当C环为二氢呋喃环时，由于氧原子上的孤对电子不参与共轭，其电负性更强，更易形成氢键，因此细胞毒性应强于C环为呋喃环的化合物。这一结论虽可用于解释二氢丹参酮Ⅰ的细胞毒性强于丹参酮Ⅰ，但却与丹参酮ⅡA细胞毒性强于隐丹参酮这一实验结果相悖，说明这其中还存在有其它的因素，如呋喃氧发挥细胞毒性作用是否仅为与DNA小沟结合，A环为脂环时对呋喃氧及二氢呋喃氧的成键是否存在影响等。因此，对于丹参酮类化合物的细胞毒性与其结构之间的关系有待更深入的研究与探讨，方能更全面地阐释其细胞毒性的分子机理，为对其进行结构改造和修饰，进而研制开发有效的创新抗癌药物提供理论与实验基础。
